# Myelopathy Secondary to Vitamin B12 Deficiency Induced by Nitrous Oxide Abuse

**DOI:** 10.7759/cureus.18644

**Published:** 2021-10-10

**Authors:** Joshua Strauss, Syed F Qadri

**Affiliations:** 1 Internal Medicine, Creighton University School of Medicine, Omaha, USA; 2 Psychiatry, Creighton University School of Medicine, Omaha, USA

**Keywords:** peripheral neuropathy, megaloblastic anemia, nitrous oxide, vitamin b12 deficiency, myelopathy

## Abstract

Nitrous oxide (N_2_O), a colorless gas known to have abuse potential, can induce vitamin B12 deficiency that eventually leads to peripheral neuropathy, central nervous demyelination, and myelopathy. N_2_O abuse has rarely caused subacute combined degeneration of the spinal cord despite being reported in a few studies. Although numerous published studies have demonstrated the toxic effects of N_2_O abuse, it is still a controversial topic of debate among neurologists. We outline a case of a patient presenting with acute onset of numbness who was ultimately diagnosed with myelopathy secondary to vitamin B12 deficiency induced by nitrous oxide abuse. This case report emphasizes the early diagnosis and management of vitamin B12 deficiency to prevent the severe complications associated with it.

## Introduction

Nitrous oxide (N_2_O) is a colorless gas known to have abuse potential and its abuse is widely recognized as a serious occupational hazard [1]. Recent research suggests that recreational usage of N_2_O has been increasing enormously because of its relaxing and euphoric effects and hallucinogenic properties [2]. N_2_O abuse or occupational N_2_O exposure can cause vitamin B12 deficiency that eventually leads to peripheral neuropathy, central nervous demyelination, myelopathy, and subacute combined degeneration along with various psychiatric effects, such as psychosis and mood changes [3].

## Case presentation

A 45-year-old male with no significant past medical or psychiatric history presented with sudden onset of numbness below the umbilicus and unsteady gait. At the time of admission, the patient also had cognitive impairment with a Montreal cognitive assessment (MoCA) score of 22/30. There was no associated phonophobia or photophobia. During the hospital stay, the patient had experienced urinary retention with subsequent Foley catheterization. On physical examination, he had no vibratory sensation or proprioception in bilateral (B/L) lower extremities with cold temperature and spared pinprick. Reflexes were 2+ in B/L knees and ankle (left) with non-sustained clonus on left ankle, big toes were down going, with diminished sensation around T10, increased muscle tone in B/L lower extremities, and no obvious muscle weakness in lower extremities. He had a normal anal tone and post-void residual. Findings were suggestive of a spinal cord lesion mainly affecting the posterior column.

All laboratory findings were unremarkable except mean corpuscular volume of 111 fl (80-100 fl), B12 level of 311 pg/mL (160-950 pg/ml), methylmalonic acid (MMA) level of 7397 nmol/L (< 370 nmol/L), and a homocysteine level of 89.7 umol/L (< 15 umol/L). He had a recent history of abuse of nitrous oxide due to recreational usage. Magnetic resonance imaging (MRI) C spine (with and without contrast), T spine (with and without contrast), L spine (with and without contrast), and MRI brain (with and without contrast) were unremarkable. Cerebrospinal fluid (CSF) analysis revealed the presence of immunoglobulin G (IgG) oligoclonal bands. Electromyography showed left non-localizable peroneal neuropathy with a small peroneal compound muscle action potential (CMAP) and small superficial peroneal CMAP with no evidence of polyneuropathy. Based on all these findings, the final diagnosis of “myelopathy secondary to vitamin B12 deficiency induced by nitrous oxide abuse” was made.

Later, the patient was treated with intramuscular vitamin B12 followed by oral formulation, and discharged to a rehabilitation center. Upon follow-up after 16 weeks, the patient's memory loss and confusion had improved with repeat B12 and MMA values of 1409 pg/mL and 139 nmol/L, respectively. The patient was able to ambulate without the assistance of a walking device and was able to perform all other activities of daily living. The patient's only remaining symptom was mild vibratory sensation deficits below the knees B/L, which was no longer noted at his 24-week follow-up.

## Discussion

Here, we presented a case of a 45-year-old man with myelopathy who heavily abused N_2_O with no significant neurological history. N_2_O is commonly used to induce general anesthesia, especially by dentists [3]. Recent research reports that 4.7% of adults use N_2_O among which young adults and adolescents have a lifetime prevalence ranging from 2% to 16% of potential abuse [3]. Established literature found that there is a link between N_2_O abuse and vitamin B12 deficiency. Accordingly, Hakimoglu et al. demonstrated that patients who received general anesthesia with N_2_O had lower postoperative serum vitamin B12 levels, compared to preoperative levels [4]. Common neurological sequelae associated with N_2_O include numbness, paresthesia, and weakness along with less common symptoms, such as bowel and bladder dysfunction, usually with a subacute onset [3]. Nonetheless, our patient differed in so far as symptoms presented in an acute fashion while driving. As mentioned in the established literature, our patient also recovered with the administration of vitamin B12 therapy over a course of weeks to months.

**Figure 1:**
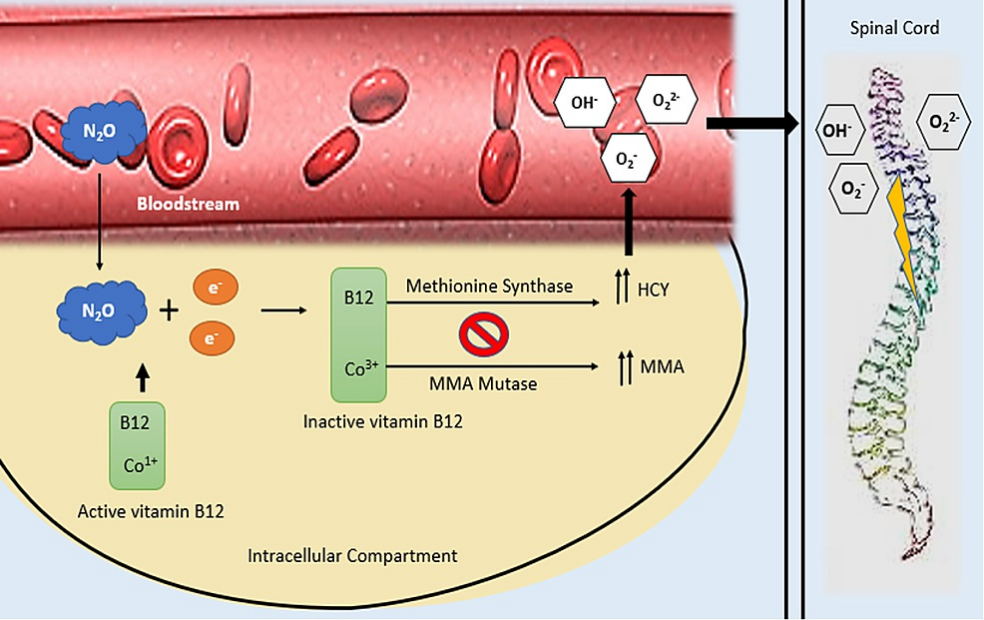
Mechanism of N2O-induced vitamin B12 depletion and subsequent myelopathy. N_2_O oxidizes the active cobalt atom (1+) of vitamin B12 to inactive cobalt form (3+), producing inactive methylcobalamin (a cofactor of methionine synthase and the active form of intracellular vitamin B12). Eventually, this process inhibits the methionine synthase enzyme-induced conversion of homocysteine to methionine and methyltetrahydrofolate to tetrahydrofolate. The proposed mechanism for myelopathy is glial cell dysfunction secondary to B12 depletion, with subsequent demyelination of the spinal cord. HCY, homocysteine; MMA, methylmalonic acid; N_2_O, nitrous oxide.

Vitamin B12 is a water-soluble vitamin that plays a pivotal role in several biochemical reactions including the generation of tetrahydrofolate (THF) in purine and pyrimidine synthesis as well as the conversion of methylmalonyl coenzyme A (CoA) (MMA) to succinyl CoA [5]. Consequently, vitamin B12 deficiency is hallmarked by megaloblastic anemia as a result of deficient DNA/RNA synthesis in erythrocytes and a buildup of MMA due to insufficient MMA mutase activity. Thus, this process eventually inhibits the methionine synthase enzyme and synthesis of methionine (Figure 1), which is a precursor of S-adenosylmethionine, a substrate necessary for myelin production, and tetrahydrofolate, which is essential for DNA synthesis [5]. Also, vitamin B12 depletion leads to a proliferation of homocysteine (HCY) due to N20 being a potent inhibitor of methionine synthetase, which in turn leads to a hypercoagulable state, increasing the likelihood of an ischemic stroke with subsequent neurological sequelae [6].

Consequentially, various neurological manifestations of B12 deficiency can occur such as loss of anal and detrusor tone as well as erectile dysfunction [4]. Psychiatric complications, such as mania, Alzheimer’s disease, confusion, memory loss, and psychosis have also been reported with N_2_O abuse [5]. N_2_O specifically oxidizes B12 rapidly leading to its irreversible inactivation and inability to be utilized in cellular maintenance of the nervous system [7]. Though many utilize N_2_O recreationally with no adverse symptoms, here, we outline a 45-year-old man with myelopathy who heavily abused N_2_O with no significant neurological history.

In the case of N_2_O-induced myelopathy, a high percentage of case reports reported that there was an increased T2 signal intensity on MRI, most often involving three or more spinal segments with a predilection for posterior cord involvement [3,8]. Similarly, our patient showed increased T2 hyperintensity in the axial view without a corresponding finding in the sagittal view. Other supporting evidence helped in confirming the diagnosis of acute myelopathy includes the presence of IgG oligoclonal bands on CSF analysis, like those seen in multiple sclerosis [3,7,8]. Our patient showed elevated MMA, homocysteine with an increased MCV indicating macrocytic anemia. Vitamin B12 depletion is also known to cause cognitive deficits. Our patient’s deficits were predominantly related to memory issues, which resulted in an ability to recall 5/5 words on MoCA.

Interestingly, the prevalence of N_2_0-induced myelopathy is higher in younger males, indicating an increased need to screen this population when discussing a patient’s social history [3]. As there is widespread usage of N_2_O, so it is becoming increasingly important to warn people, especially those of younger age, about the potential harms this substance may cause. Thus, early interventions, such as drug abstinence programs, would be crucial in preventing poor outcomes. Though prevention will not always prevail, this case also demonstrates the necessity for thorough social history to address the full extent of the abuse. Also, given our patient’s history of veganism, his B12 was most likely depleted predisposing him to a vitamin B12 deficiency [1], further highlighting the importance of initial history and physical examination. With regards to therapeutics, prompt administration of vitamin B12 is key to abating any potential permanent or long-term neurological sequelae which might arise. It is also pivotal to continuously monitor a patient’s mental status with serial cognitive assessments over time.

## Conclusions

N_2_O abuse can lead to vitamin B12 deficiency, resulting in neurologic sequelae characterized by the presence of elevated methylmalonic and homocysteine levels. Healthcare professionals should suspect possible N_2_O abuse in young adults with neurologic manifestations of B12 deficiency in case of no other alternative etiologies. Cessation of N_2_O use along with supplementation of vitamin B12 would be beneficial in the complete resolution of symptoms.
